# The small vesicular culprits: the investigation of extracellular vesicles as new targets for cancer treatment

**DOI:** 10.1186/s40169-017-0176-z

**Published:** 2017-12-13

**Authors:** Fumihiko Urabe, Nobuyoshi Kosaka, Yusuke Yoshioka, Shin Egawa, Takahiro Ochiya

**Affiliations:** 1 Division of Molecular and Cellular Medicine, National Cancer Center Research Institute, 5-1-1 Tsukiji, Chuo-ku, Tokyo, 104-0045 Japan; 2 Department of Urology, Jikei University School of Medicine, 3-19-18 Nishi-Shimbashi, Minato-ku, Tokyo, 105-8471 Japan

**Keywords:** Extracellular vesicles, miRNA, Clinical application

## Abstract

Extracellular vesicles (EVs) are membranous vesicles released from almost all type of cells including cancer cells. EVs transfer their components, such as microRNAs (miRNAs), messenger RNAs, lipids and proteins, from one cell to another, affecting the target cells. Emerging evidence suggests that reciprocal interactions between cancer cells and the cells in their microenvironment via EVs drive disease progression and therapy resistance. Therefore, understanding the roles of EVs in cancer biology will provide us with new opportunities to treat patients. EVs are also useful for monitoring disease processes. EVs have been found in many kinds of biological fluids such as blood, urine, saliva and semen. Because of their accessibility, EVs offer ease of collection with minimal discomfort to patients and are preferred for serial collection. In addition, they reflect and carry dynamic changes in disease, allowing us to access crucial molecular information about the disease status. Therefore, EVs hold great possibility as clinically useful biomarkers to provide multiple non-invasive snapshots of primary and metastatic tumors. In this review, we summarize current knowledge of miRNAs in EVs in cancer biology and as biomarkers. Furthermore, we discuss the potential of miRNAs in EVs for clinical application.

## Introduction

Intercellular communication plays an essential role in multicellular organisms and can be mediated through direct cell–cell contact or through the transfer of secretory molecules. Recent studies have shown that as a new intercellular communication mechanism, small lipid bilayer vesicles, termed extracellular vesicles (EVs), play key roles in cancer progression and have great potential in clinical applications.

**Fig. 1:**
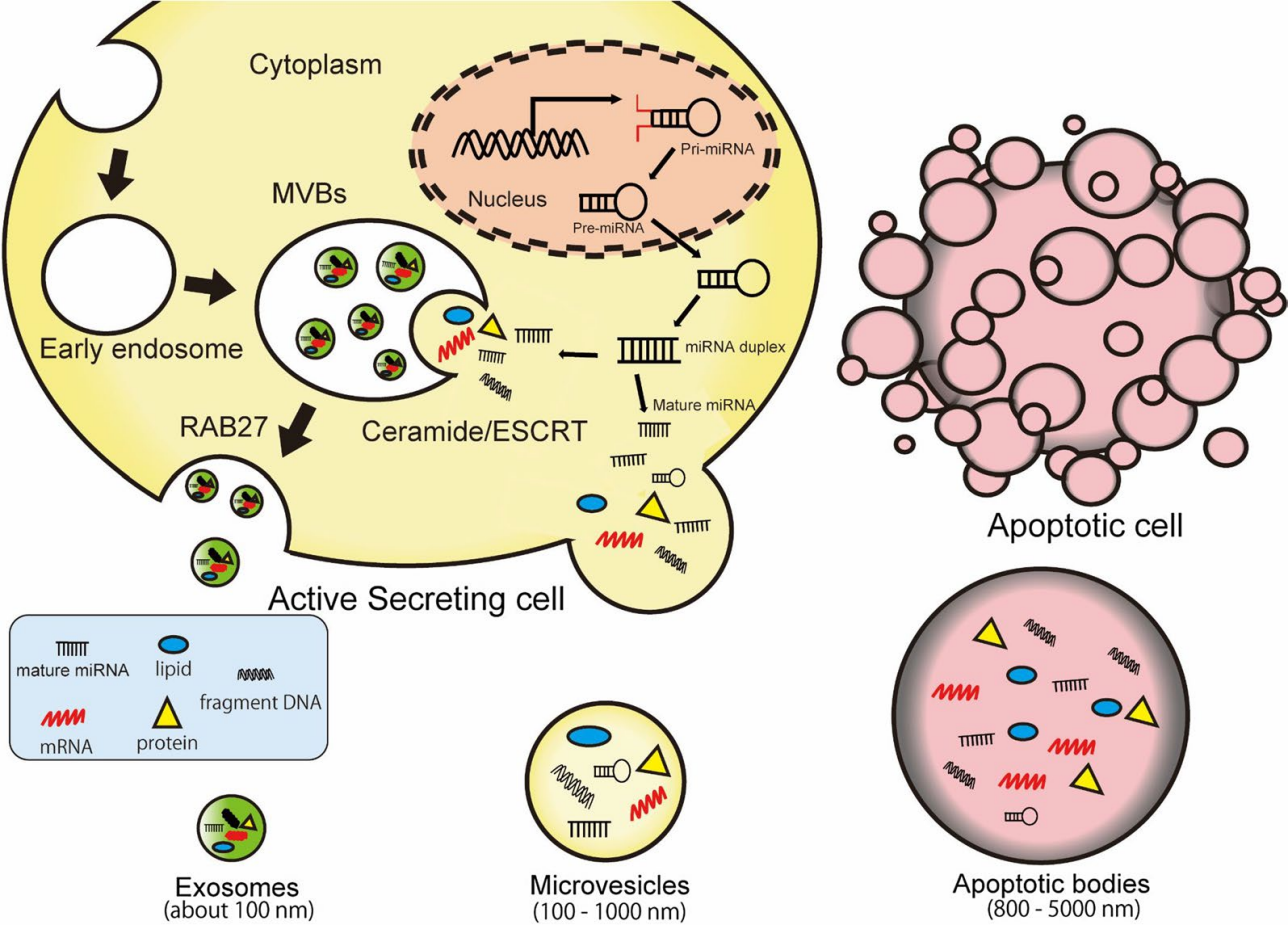
The classification of extracellular vesicles. EVs contain various molecules, such as miRNAs, mRNAs, DNAs, proteins and lipids. Exosomes are formed by inward budding into early endosomes to form MVBs. This inward budding process involves ceramide-dependent mechanisms or an ESCRT. Subsequently, these MVBs fuse with the limiting plasma membrane to release exosomes into the extracellular space. This fusion process is dependent on Rab GTPases (e.g., Rab27A/B). MVs are directly shed or bud from the plasma membrane. Apoptotic bodies are released from the cell undergoing programmed cell death. Pri-miRNA: primary microRNA

EVs are heterogeneous populations of vesicles that are secreted by almost all types of cells [1]. EVs include exosomes, microvesicles and apoptotic bodies, and these subgroups are categorized according to their origin, size and properties [2] (Fig. 1). Exosomes are small EVs (approximately 100 nm) and are derived from the intra-cellular endosomal compartment. Exosomes are initially formed by a process of inward budding into early endosomes to form multivesicular bodies (MVBs). These MVBs fuse with the limiting plasma membrane to release exosomes into the extracellular space [3, 4]. As exosomal markers, members of the tetraspanin family (CD9, CD63 and CD81), members of the endosomal-sorting complex required for transport (ESCRT) complex (TSG101, Alix), heat shock proteins (Hsp60, Hsp70, Hsp90) and Rab proteins (Rab27A/B) are recognized [5, 6]. Microvesicles are larger than exosomes (100–1000 nm) and are directly shed or bud from the plasma membrane in response to stimulation [7]. Microvesicles have been reported to be enriched in phosphatidylserine and have several lipids components [8]. Apoptotic bodies are several micrometers in diameter (800–5000 nm) and are released from the cell undergoing programmed cell death. Despite being classified by the origin of these vesicles, we must consider that current techniques cannot clearly distinguish each type of EV separately [9]. To avoid confusion, in this review, we use EVs as a general term for all types of vesicles in the extracellular milieu, according to the recommendation of the International Society for Extracellular Vesicles (ISEV) [10].

Although EVs have long been considered as disposal vehicles to eliminate unwanted proteins and biomolecules [11], in 2007, Valadi et al. identified miRNA as well as mRNA inside EVs and showed the potential functionality of these nucleic acids in recipient cells [12]. miRNAs are small single-stranded non-coding RNAs that negatively regulate gene expression by binding to the 3′ untranslated region (3′ UTR) of mRNA, leading to mRNA degradation or the inhibition of translation [13]. Through this mechanism, miRNAs are involved in the progression of various diseases including cancer [14]. In 2010, three independent groups published that miRNAs in EVs can be transferred to immune cells [15], cancer cells [16], and endothelial cells [17] and have functions within these cells. Subsequently, many researchers started to focus on miRNAs in EVs, and accumulating evidence has demonstrated that EVs transfer miRNAs from one cell to another and that their components have an effect on cancer progression [18, 19].

In this review, we summarize the potential of miRNAs in EVs in clinical applications. First, we describe the contribution of miRNAs in EVs to cancer biology. Next, we discuss the possibility of EV-targeting therapy. In the second part, we provide an overview of the utility of miRNAs in EVs as biomarkers of several major cancers. We focus on the problem associated with the present tumor biomarkers and discuss the possible uses of miRNAs in EVs as diagnostic and prognostic biomarkers.

## The role of EVs in the cancer microenvironment

### Induction of angiogenesis and endothelial cell permeability

Due to the hypoxic condition in tumors, angiogenesis, the formation of new blood vessels from an existing vasculature, is crucial for cancer cells [20]. Abnormal tumor angiogenesis is widely accepted as the major problem in cancer due to its contribution to cancer proliferation and therapy resistance. In addition, tumor vessels exhibit a high permeability, increasing metastatic dissemination [21, 22]. Endothelial cells are one origin of tumor vessels [23], and accumulating evidence has shown that cancer cell communication with endothelial cells via miRNAs in EVs is associated with angiogenic activity in tumors [24–27].

For example, Zhuang et al. reported that miR-9-5p in EVs from murine cancer cells was delivered into endothelial cells and promoted the migration of endothelial cells and angiogenesis. They found that secreted miR-9-5p reduced levels of suppressor of cytokine signaling 5 (SOCS5) and activated the JAK-STAT pathway in endothelial cells, leading to angiogenesis in tumors [24]. miR-210 is described as the major hypoxamir [28], inducing angiogenesis in endothelial cells and targeting Ephrin-A3 [29]. Several reports have shown that miR-210-3p in EVs also contributes to tumor angiogenesis [25, 26]. Kosaka et al. showed that the neutral sphingomyelinase 2 (nSMase2) regulates EV-associated miR-210-3p secretion and promotes angiogenesis, which affects the capacity for metastasis [25]. Cui et al. reported that tissue inhibitor of metalloprotease-1 (TIMP-1) lead to an accumulation of miR-210-3p in EVs via activation of the PI3K/AKT/HIF-1/miR-210 signaling cascade under normoxic conditions [26]. In another study, Hsu et al. have shown that miR-23a-3p in EVs from hypoxic lung cancer cell increase not only angiogenesis but also vascular permeability. EVs containing miR-23a-3p directly suppress prolyl hydroxylase 1 and 2, activating the expression of hypoxia-inducible factor-1α in endothelial cells, which results in angiogenesis induction. miR-23a-3p also inhibits the tight junction protein zonula occludens-1 (ZO-1), which has been reported to play a central regulatory role in controlling angiogenesis barrier formation [30] and thereby increasing vascular permeability [27].

Tumor-derived EVs affect endothelial cells in distant microenvironments to form a metastatic site. The blood–brain barrier (BBB) consists of endothelial cells and surrounding cells, and it normally serves as a defensive barrier. BBB destruction is one of the key events during brain metastasis [31], and previous reports have shown that miRNAs in cancer-derived EVs increase endothelial cell permeability and induce brain metastasis. Zhou et al. showed that metastatic breast cancer-derived EVs contain miR-105-5p, which targets the tight junction protein ZO-1, thereby inducing endothelial cell permeability and brain metastasis [32]. In another study, Tominaga et al. showed that EVs derived from a brain metastatic breast cancer cell line, which contains miR-181c-5p, induce destruction of the BBB [33]. miR-181c-5p negatively regulates 3-phosphoinositide-dependent protein kinase-1 (PDPK1) and causes degradation of phosphorylated cofilin and severing of actin filaments [33]. Thus, cancer-derived EVs regulate the endothelial cell phenotype, thereby contributing to cancer progression and metastasis. From a clinical perspective, sunitinib and sorafenib, which are popular molecular target drugs, inhibit the effect on vascularization by targeting vascular endothelial growth factor receptors (VEGFRs) or platelet-derived growth factor receptors (PDGFRs) [34]. However, previous studies have reported that these drugs are not successful in establishing a beneficial effect in advanced lung and breast cancer [35, 36]. As previously mentioned, the transfer of miRNAs in EVs derived from lung and breast cancer cells to endothelial cells activate different pathways to regulate angiogenesis or vascular permeability, which could be targeted by vascularization-inhibiting molecular drugs [24–27]. Therefore, intercellular transfer of EVs could be a new therapeutic target, especially for lung and breast cancer.

### Crosstalk between cancer cells and stromal fibroblasts

Cancer-associated fibroblasts (CAFs) are the major component of the tumor microenvironment, and CAFs have been reported to play a key role in malignant progression [37].

The mechanism responsible for CAF induction remains controversial, but recent studies have shown that TGF-β is partially responsible for activating CAFs [38, 39]. In addition, EVs derived from cancer cells also induce CaF-like phenotypes in resident fibroblasts. Pang et al. showed that EVs derived from pancreatic cancer containing miR-155-5p, which targets TP53INP1, result in the proliferation and activation of normal fibroblasts [40].

In contrast, CAFs also provide a benefit for cancer progression. Yeung et al. demonstrated that CAFs and cancer-associated adipocytes secrete higher levels of miR-21-5p in EVs than in those from ovarian cancer cells, by using next-generation sequencing technology. In that study, they also revealed that miR-21-5p suppresses ovarian cancer apoptosis and confers chemoresistance by binding to AFAP1 [41]. Donnarumma et al. showed that in breast cancer, CAF-derived EVs contain three miRNAs (miR-21-5p, -378e, 143-3p) that induce stemness and epithelial–mesenchymal transition (EMT) of breast cancer cells, regulating the development of an aggressive cancer phenotype [42].

In another study, Baroni et al. observed cross-talk between cancer cells and fibroblasts. miR-9-5p, which is upregulated in triple-negative breast cancer (TNBC)-derived EVs, induces CAF-like properties in human breast fibroblasts. Moreover, fibroblasts activated by miR-9-5p alter the tumor behavior, modulating genes involved in cell motility and extracellular matrix (ECM) modeling [43]. Thus, interactions between cancer cells and CAFs via EVs promote cancer proliferation.

In addition, several reports have revealed that cancer-derived miRNAs in EVs contribute to cancer progression through remodeling the fibroblasts within the distant site. Rana et al. found that cancer-associated miR-494 and miR-542-3p in EVs are transferred to lymph node stromal cells and lung fibroblasts, targeting cadherin-17 and upregulating matrix metalloprotease [44]. Fong et al. revealed that breast cancer cell-derived miR-122-5p in EVs suppresses glucose intake in astrocytes and lung fibroblasts by inhibiting pyruvate kinase. This increased glucose utilization and promotion of circulating tumor cell colonization favors brain and lung metastasis [45]. Fibroblast remodeling at distant sites is one of the major components of the developing premetastatic niche [46], and these studies indicate an additional contributory role of miRNAs in EVs.

### Modulation of the immune system

Escape from immune-mediated tumor destruction has been recognized as a hallmark of cancer [47]. Zitvogel et al. and Wolfers et al. were the first researchers to show the relationship between EVs derived from cancer cells and the immune system, and many studies have since confirmed this relationship [48, 49]. In the tumor microenvironment, immune cells, such as macrophages, natural killer cells, T lymphocytes and B lymphocytes, interact with tumor cells and regulate tumorigenesis and progression. It has been acknowledged that tumor progression is partly related to the extent of immune dysfunction, and emerging data indicate that EVs are a novel contributor to the immune modulation. EVs derived from cancer mostly have immunosuppressive effects that support tumor progression and metastasis. For instance, Kim et al. have shown that EVs from oral cancer patient sera contain FasL and induce apoptosis in Jurkat and CD8^+^ T cell [50]. Myeloid-derived-suppressor cells (MDSCs) have been identified as a population of immature myeloid cells with the ability to suppress T cell activation, contributing to cancer proliferation [51]. Chalmin et al. found that heat shock protein 72 (Hsp72) expressed on tumor-derived EVs triggers STAT3 activation in MDSCs through toll-like receptor 2, inducing their immunosuppressive activity [52].

In addition, cancer cell-derived EVs recruit immune cells to enhance tumor invasion and dissemination. Fabbri et al. have revealed that lung cancer-derived EVs containing miR-21-5p and miR-29a-3p, which bind as ligands to the toll-like receptor (TLR) family (murine *TLR7* and human TLR8) in surrounding tumor-associated macrophages (TAMs), trigger NF-κB-mediated pro-inflammatory cytokine production and support the progression of cancer [53]. In another study, Ying et al. have found that miR-222-3p in EVs derived from epithelial ovarian cancer (EOC) cells shift macrophages toward a tumor-supportive TAM-like phenotype [54]. Regarding the immune escape system, many types of cancer cells upregulate the expression of programmed death-1 ligand (PD-L1), which plays an important role in blocking the immune system by binding to PD-1 expressed on the surface of T cells and induces programmed death in activated T cells [55]. Several studies have revealed that miRNA indirectly regulates the expression of PD-L1 in cancer cells. Fujita et al. demonstrated that miR-197-3p indirectly regulates PD-L1 expression via the miR-197/CKS1B/STAT3-mediated PD-L1 network [56]. Chen et al. showed that the expression of PD-L1 in lung cancer is regulated by the miR-200/ZEB1 axis and the subsequently suppresses CD8^+^ T cells in the tumor environment [57]. In 2016, Kataoka et al. reported that disruption of the PD-L1 3′-untranslated region (UTR) is associated with cancer cells aberrantly expressing PD-L1 [58]. The 3′ UTR is the site bound by miRNAs, suggesting the possibility that miRNAs may directly mediate the expression of PD-L1. In addition, Haderk et al. recently reported that noncoding Y RNA hY4 in EVs derived from chronic lymphocytic leukemia (CLL) modulate PD-L1 expression in monocytes [59]. Although confirmation is still needed, these results support the presence of miRNAs packaged in EVs to regulate PD-L1 expression.

### Regulation of cancer cell proliferation and drug resistance

EVs derived from cancer cells or microenvironmental cells affect cancer cell proliferation and drug resistance and regulate tumor progression during various phases.

First, during tumor initiation, there is competition between cancer cells and the surrounding normal epithelial cells [60]. Kosaka et al. demonstrated that normal epithelial prostate cells secrete EVs containing miR-143-3p, suppressing the proliferation of prostate cancer cells [16]. miR-143-3p in EVs derived from normal epithelial prostate cells negatively regulates KRAS and ERK5, repressing the proliferation of cancer proliferation. Normal epithelial cells derived EVs contribute to the maintenance of homeostasis and prevent cancer initiation; however, once the cancer cells overcome the suppression, the primary tumor starts to progress.

Primary tumors consist of heterogeneous cells with varying proliferative, invasive and metastatic abilities. Hence, through the intra-tumor transfer of EVs, tumor cells can collaborate to drive tumor progression. Le et al. showed that the transfer of miR-200 family from metastatic breast cancer cells to poorly metastatic breast cancer cells promote mesenchymal-to-epithelial transition (MET) [61]. Although metastasis involves multiple steps, MET is a crucial step during the development of metastasis at distant sites. In a xenograft model, they revealed that miR-200 miRNAs in EVs from metastatic cells promoted metastasis in otherwise weakly metastatic cells, and demonstrated that the metastatic capacity could be transferred via the uptake of EVs. In another study, Singh et al. reported that the transfer of miR-10b-5p in EVs from metastatic breast cancer cells promotes the invasive capacity of non-malignant cells by targeting HOXD10 [62]. Although their findings did not directly indicate that less invasive cancer cells became more invasive via the transfer of EVs from metastatic cancer cells, their results suggest that cancer-associated miRNAs in EVs can promote adjacent cells and lead to outcomes favoring tumor proliferation.

For patients with advanced-stage cancer, chemotherapy and targeted drugs are the main treatment strategies; however, their effectiveness does not last for long periods due to resistance [63]. Several studies have shown that EVs play a role as a noteworthy vehicle of the dissemination of cancer drug resistance. Indeed, horizontal transfer of miRNA via EVs is one of the main mechanisms leading to drug resistance. Chen et al. revealed that drug-resistant breast cancer cells secrete several miRNAs (miR-30a-5p, miR-100-5p and miR-222-3p) that are enriched in EVs, reducing the drug sensitivity of drug-sensitive cancer cells [64]. Recently, Wei et al. indicated that miR-222-3p, which is secreted by drug-resistant non-small-cell lung cancer (NSCLC) cells in EVs, is transferred and promotes gemcitabine resistance in sensitive cells by targeting suppressor of cytokine signaling 3 (SOCS3) [65]. Challagundla et al. revealed that a unique cross-talk between neuroblastoma cells and human monocytes through miR-21-5p and miR-155-5p in EVs contributes to the development of drug resistance. NBL cells secrete miR-21-5p in EVs transferred to monocytes, activating the NF-κB pathway and upregulating miR-155-5p in monocytes. In return, activated monocytes secrete miR-155-5p in EVs transferred to NBL cells, targeting TERF1 and providing drug resistance [66].

EVs are also related to long-term recurrence. Bone marrow is one of the major homing organs for disseminated breast cancer cells. Even 10–20 years after resection of the primary site, breast cancer patients often develop a recurrence, especially in the bone marrow [67], which indicates that breast cancer cells spread and survive for a long time in a dormant state. Ono et al. revealed that the mechanism responsible for the maintenance of dormancy in bone marrow includes the transfer of miRNA in EVs secreted by bone marrow mesenchymal stem cells (BM–MSC) [68]. miR-23b-3p in BM–MSC-derived EVs contribute to the dormant state of breast cancer cells by downregulating a target gene, myristoylated alanine-rich C-kinase substrate (MARCKS), which encodes a protein that promotes cell cycling a motility. Therefore, cancer surrounding noncancerous BM–MSCs plays an important role in inducing breast cancer cells dormancy and future recurrence.

As summarized above, EVs have various effects on cells in the cancer microenvironment and on cancer cells themselves, participating in the various timing features of tumor progression. Thus, it is crucial to understand the molecular mechanisms underlying cancer progression by EVs, and then we can consider tactics to defeat cancer by targeting EVs. In the next section, therapeutic strategies by EV targeting are discussed.

### EV-targeting therapeutic strategies

**Fig. 2:**
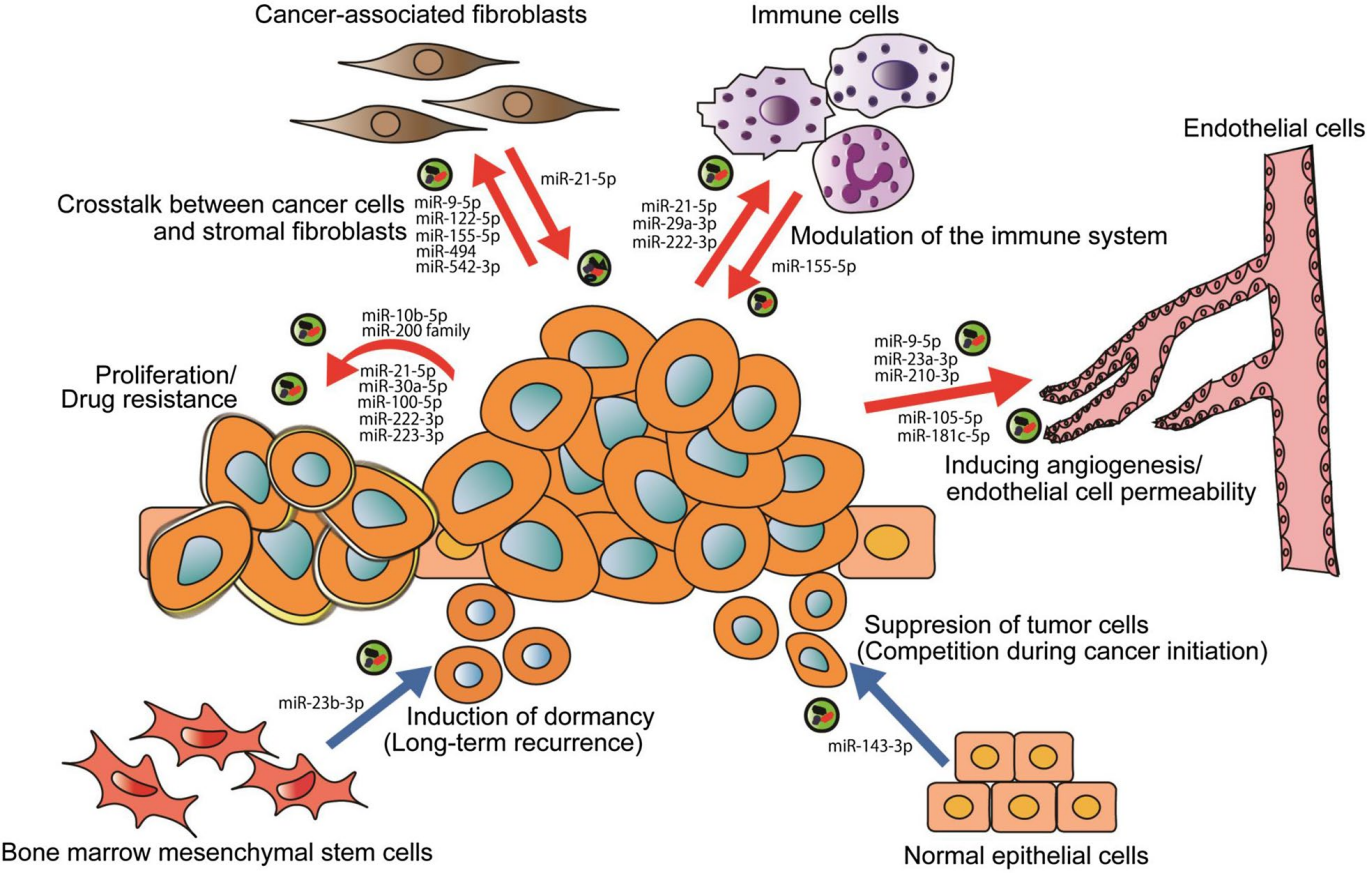
Role of miRNAs in EVs in the cancer microenvironment. Through the transfer of miRNAs, EVs mainly promote tumorigenesis. Tumor-derived EVs can activate endothelial cells to promote angiogenesis or vascular permeability. Tumor-derived EVs can convert fibroblasts into CAFs. In return, CAF-derived EVs can confer proliferation or drug resistance to tumor cells. Tumor-derived EVs can contribute to creating an immunosuppressive microenvironment by impairing the function of immune cells. In another aspect of the immune system, tumor-derived EVs can mobilize TAMs to promote cancer progression. Moreover, tumor-derived EVs can provide drug resistance or proliferation to surrounding other tumor cells. In contrast, noncancerous cell-derived EVs also participate in the tumor microenvironment. EVs derived from MSCs contribute to long-term metastasis by inducing a dormant state in cancer cells. EVs derived from normal epithelial cells can suppress cancer proliferation

**Fig. 3:**
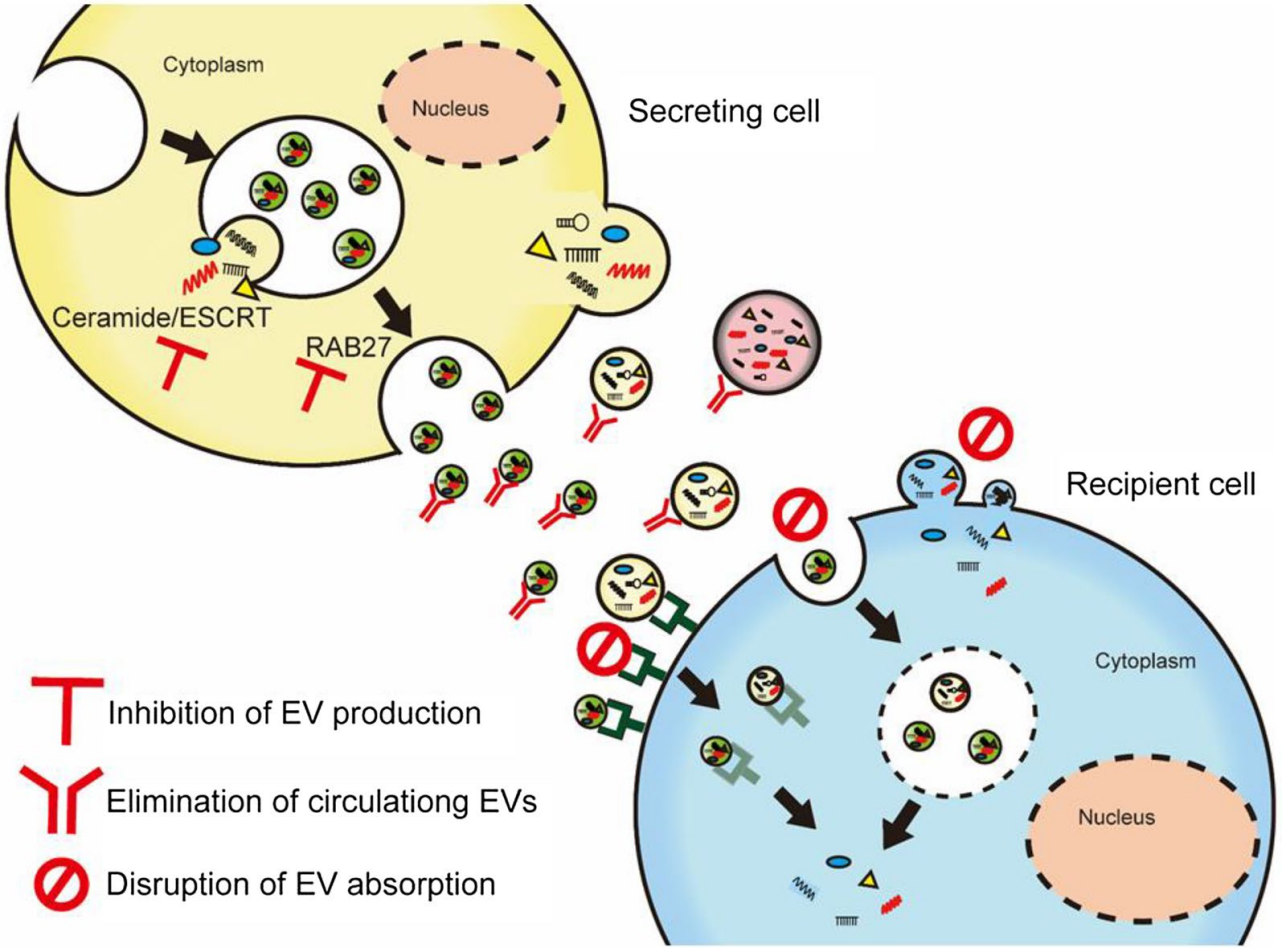
Schematic of EV-targeting therapy. Intercellular transfer of the EV cargo contributes to cancer development, so reducing EV transfer will provide new therapeutic strategies. Inhibition of EV production, elimination of circulating EVs and disruption of EV absorption will be main strategies

As we have shown, intercellular communication via EVs contributes to tumor progression through the transfer of their cargo (Fig. 2; Table 1). In addition, the pathways that are activated by the transfer of miRNAs in EVs mostly differ from those targeted by modern drugs, such as chemotherapy or molecular targeting drugs. Therefore, a reduction of cancer-derived EVs transfer will provide additional therapeutic value for inhibiting cancer proliferation and dissemination. Three potential therapeutic strategies, inhibition of EV production, elimination of circulating EVs and disruption of the absorption of EVs, have been proposed [18] (Fig. 3). In this section, we summarize these strategies and discuss their potential clinical applications.

**Table 1:** miRNAs in EVs in cancer microenvironment

Category	Function	miRNAs	Target genes	EV producing cells	Recipient cells	Refs.
Inducing angiogenesis and endothelial cell permeability	Enhancement of angiogenesis	miR-9-5p	SOCS5	Melanoma cells	Endothelial cells	[24]
	Enhancement of angiogenesis	miR-210-3p	EFNA3	Lung cancer cells	Endothelial cells	[25, 26]
	Enhancement of angiogenesis and vascular permeability	miR-23a-3p	PHD 1 and 2, ZO-1	Lung cancer cells	Endothellial cells	[27]
	Destruction of BBB	miR-105-5p	ZO-1	Breast cancer cells	Endothelial cells (distant site)	[32]
	Destruction of BBB	miR-181c-5p	PDPK1	Breast cancer cells	Endothelial cells (distant site)	[33]
Crosstalk between cancer cells and stromal fibroblasts	Induction of CAFs	miR-155-5p	TP53INP1	Pancreastic cancer cells	Fibroblasts	[40]
	Suppression of apoptosis and confering chemoresistance	miR-21-5p	AFAP1	CAFs and CAAs	Breast cancer cells	[41]
	Stemness and EMT	miR-21-5p, miR-378e and miR-143-3p	Not mentioned	CAFs	Breast cancer cells	[42]
	Induction of CAFs	miR-9-5p	Not mentioned	Breast cancer cells	Fibroblasts	[43]
	Development of premetastatic niche	miR-494 and miR-542-3p	CDH17	Metastatic rat pancreatic adenocarcinoma	Lymph node stromal cells and lung fibroblasts (distant site)	[44]
	Development of premetastatic niche	miR-122-5p	PKM2, CS	Breast cancer cells	Astrocytes and lung fibroblasts (distant site)	[45]
Modulation of immune system	Induction of TAMs	miR-21-5p and miR-29a-3p	Directly bind to TLR family (murine *TLR7* and human TRL8)	Lung cancer cells	Murine macrophages	[53]
	Induction of TAMs	miR-222-3p	SOCS3	EOC cells	Monocytes	[54]
Regulation of cancer cell proliferation and drug resistance	Supression of cancer cell proliferation (competition during cancer initiation)	miR-143-3p	KRAS and ERK5	Normal prostate epithelial cells	Prostate cancer cells	[16]
	Promoting MET	miR-200 family	ZEB2, SEC23A	Highly metastatic lung cancer cells	Weakly metastatic lung cancer cells	[61]
	Promoting invasiveness	miR-10b-5p	HOXD10	Metastatic breast cancer cells	Non-malignant breast epithelial cells	[62]
	Adriamycin and docetaxel resistance	miR-30a-5p, miR-100-5p and miR-222-3p	PTEN (miR-222)	Drug resistant breast cancer cells	Drug sensitive breast cancer cells	[64]
	Gemcitabine resitance	miR-222-3p	SOCS3	Drug resistant NSCLC cells	Drug sensitive NSCLC cells	[65]
	Activating monocytes	miR-21-5p	Directly bind to TRL8	Neuroblastoma cells	Human monocytes	[66]
	Cisplatin resistance	miR-155-5p	TERF1	Activated monocytes	Neuroblastoma cells	[66]
	Induction of dormant state (long-term recurrecnce)	miR-23b-3p	MARCKS	BM–MSCs	Breast cancer cells	[68]

Several articles have described the effectiveness of inhibiting EV production in vitro and in vivo. In 2013, Kosaka et al. revealed that knockdown of nSMase2, which is required for the synthesis of ceramide, EV secretion and miR-210-3p transfer, is inhibited, and angiogenesis and metastasis in a xenograft mouse model are suppressed [25]. In another study, Yokoi et al. reported that knockdown of nSMase2 suppresses peritoneal dissemination in ovarian cancer by inhibiting EV production [69]. Until now, other molecules related to EV production, such as RAB27A, RAB27B and TSG101, have also been reported to be effective for inhibiting cancer-derived EV production [70, 71]. Inhibition of EV production will provide a chance to suppress intercellular communication, and therefore this strategy has great potential for the treatment of cancer. However, these genes have key roles in multiple cell biological events, and therefore their downregulation in normal cells would have adverse effects on normal cell functioning [18]. Indeed, it has been reported that nSMase2 is expressed in normal neural cells [72]. In addition, the downregulation of these genes may not have the same inhibitory effects on EV secretion between cancer types. Phuyal et al. have reported that inhibition of nSMase2 does not inhibit EV secretion in a prostate cancer cell line [73]. Therefore, to identify the genes related to cancer type-specific EV production is a future challenge.

In 2012, Marleau et al. described a therapeutic strategy for the removal of circulating EVs. In their study, they developed the hemofiltration system, which can specifically capture circulating cancer cell-derived HER2-positive-EVs [74]. HER2-expressing EVs have been shown to interfere with therapy and are associated with cancer progression [75]; therefore, selectively eliminating HER-2-expressing EVs could be a new strategy to treat breast cancer. In 2012, Peinado et al. showed that circulating EVs contribute to cancer metastasis by establishing a premetastatic niche, and they suggested a therapeutic strategy [76]. Thus, it is natural to focus on the possibility that targeting the circulating EVs from cancer cells could be a strategy for preventing cancer metastasis. Recently, Nishida-Aoki et al. revealed a new idea for eliminating EVs [77]. They showed that in a human breast cancer xenograft mouse model, administration of antibodies against human-specific CD9 and CD63, which are enriched on the surface of EVs, significantly decreased metastasis, although no obvious effects on primary site growth were observed. In that study, EVs tagged by anti-CD9 and CD63 were internalized by macrophages via phagocytosis before they could promote cancer progression. In humans, anti-CD9 and CD63 antibodies cannot selectively attach to cancer-derived EVs, so further investigations are needed. However, if we can identify the location of the cancer-specific molecules on EVs in more detail, circulating EV elimination, via using their antibodies, could potentially be applied to treat patients. Therefore, that study suggested a new novel treatment strategy for cancer.

Inhibition of EV internalization will also provide new therapeutic strategies. Christianson et al. showed that heparan sulfate proteoglycans (HSPGs) serve as receptors of EVs derived from glioblastoma (GBM) [78]. Heparin, which is an HS mimetic, dose-dependently inhibits EV uptake and suppresses EV-dependent cell migration in GBM. Several other molecules related to EV internalization have also been reported [79–81]. However, the mechanism responsible for EV internalization is quite complex and more obscure than the secretion mechanism. Unfortunately, no study has demonstrated an effect of inhibiting EV internalization in vivo. One explanation for this deficit is the lack of detailed knowledge about the cancer cell-specific EV uptake pathway. Like the other two strategies, avoiding damage to normal cell homeostasis should be a priority during therapeutic development, and thus the identification of cancer-specific EV uptake pathways is crucial. In 2016, Kowal et al. reported that different EV fractions have different EV protein markers [82], and they suggested that different EV fractions have different molecular and biological properties. Furthermore, in 2017, Tkach et al. reported that immature dendritic cells secrete two different EV subpopulations, namely, small EVs and large EVs, which have different effects on T helper cell [83]. Therefore, the identification of EV subpopulations with more oncogenic cargo that affect recipient cells and their specific internalization pathways may be the most effective strategies.

As we have shown, inhibition of EV transfer suppresses tumor development and represents a new therapeutic strategy. Although many challenges remain, the astonishing advances in the EV field provide promise that they will be overcome. In the future, targeting EV therapy would be considered a standard treatment, such as surgery, radiotherapy and chemotherapy.

### EV-associated miRNAs as biomarkers in cancer

Approximately 10 years ago, the existence of miRNA in EVs and the presence of tumor-derived EVs in the peripheral circulation were reported [12, 84]. Since then, many studies have supported the possibility of miRNAs in EVs as cancer biomarkers.

Compared with conventional tissue biopsy, EV-based liquid biopsy has several merits. First, almost all cells secrete EVs, which can be found in many kinds of body fluids such as blood [85], urine [86], saliva [87] and semen [88]. Due to their easy accessibility, EVs offer ease of collection with minimal discomfort to the patients and are preferred for serial collections. In addition, although the small size of a tissue biopsy may not reflect the total genetic heterogeneity within the disease, EVs shed from heterogeneous cancers reflect the dynamic changes that occur during disease and allow us to access crucial molecular information about the status of diseases. Furthermore, miRNAs in EVs are more suitable for the development of biomarkers than other circulating miRNAs because EVs are small lipid bilayer vesicles, and their cargo is protected from ribonucleases [3]. Therefore, EVs hold greater possibilities as clinically useful biomarkers to provide multiple non-invasive snapshots of primary and metastatic tumors. In this section, we describe the limitations of current biomarkers and summarize recent clinical sample-based studies of diagnostic and prognostic biomarkers in the six major cancers.

### Lung cancer

Lung cancer is the most common and the leading cause of cancer-related death in the United States [89]. NSCLC accounts for approximately 80% of all cases of lung cancer, and NSCLC has a poor 5-year survival rate due to the delay in detection of the disease [90]. There are no validated procedures to detect early-stage NSCLC other than low-dose helical computed tomography scanning [91], and therefore valuable biomarkers are required. Several reports have demonstrated the usefulness of miRNAs in EVs as diagnostic or prognostic biomarkers for NSCLC. In 2013, Cazzoli et al. used wide-range miRNA analysis to select candidate miRNAs in plasma-derived EVs from 10 patients with NSCLC, 10 patients with lung granuloma and 10 healthy smokers. Subsequently, selected miRNAs were validated in a larger independent group of samples (105 NSCLC patients, 50 lung granuloma patients and 25 healthy smokers). The result showed that the screening model (including miR-378a-3p, miR-379-5p, miR-139-5p and miR-200-5p) distinguishes between patients with any kind of nodules and control smokers, with an area under the receiver operating characteristic curve (AUC) of 0.908, sensitivity of 97.5% and specificity of 72.0%. The diagnostic model (including miR-151-5p, miR-30a-3p, miR-200b-5p, miR-629-5p, miR-100-5p and miR-154-3p) discriminates between NSCLC and granuloma, with an AUC of 0.76, sensitivity of 96% and specificity of 60% [92].

In 2017, Jin et al. reported highly sensitive noninvasive biomarkers for early detection of NSCLC. They performed RNA-sequence (RNA-seq) to identify candidates and validate adenocarcinoma and squamous cell carcinoma (SCC)-specific miRNAs from 46 stage I NSCLC patients and 42 healthy individuals. They detected diagnostic biomarkers for NSCLC (let-7b-5p, miR-21-5p, miR-24-3p and miR-486-5p), adenocarcinoma (miR-181-5p, miR-30a-3p, miR-30e-3p and miR-361-5p) and SCC (miR-10b-5p, miR-15b-5p and miR-320b). Surprisingly, the diagnostic accuracy of combination miRNA panels exhibited an area under the curve (AUC) value of 0.899, 0.936, and 0.911 for the detection of NSCLC, adenocarcinoma, and SCC [93].

Despite the progression of NSCLC therapy, the prognosis for patients with NSCLS remains poor. Therefore, clarification of the prognostic biomarkers is required to improve the outcome of NSCLC patients. In 2017, Liu et al. found that miR-23b-3p, miR-10b-5p and miR-21-5p in plasma-derived EV are independent prognostic biomarkers for NSCLC. They selected candidate miRNAs using a qPCR-array panel and validated by qRT-PCR. Addition of the three miRNAs significantly improved the predictive accuracy for survival, with an increase in the time-dependent AUC from 0.88 to 0.91 [94]. Dejima et al. used microarray analysis to examine plasma from 3 NSCLC patients with recurrence after surgery, 3 patients without recurrence and 3 healthy volunteers. They then assessed the candidate miRNAs in a separate cohort of 195 NSCLC patients and 30 healthy individuals. The results showed that disease-free survival was significantly worse in the high miR-21-5p group and the high miR-4257 group, respectively, suggesting that the expression of miR-21-5p and miR-4257 in EVs has potential as a predictive biomarker for recurrence after surgical resection [95].

### Colorectal cancer

Colorectal cancer (CRC) is the third most common cancer and the second leading cause of cancer-related death in the United States [89]. The prognosis of CRC is dependent on the disease stage at diagnosis, with a 5-year survival rate of 90% when diagnosed in the early stage [96]. Colonoscopy is the authorized method for diagnosis, but its invasiveness and uncomfortableness inhibit medical examination. The serum markers CA199 and CEA are useful for detecting CRC, but with low sensitivity and specificity [97]. Therefore, reliable and non-invasive biomarkers are needed.

In 2014, Ogata-Kawata et al. reported the possibility of seven miRNAs (let-7a-5p, miR-1229-3p, miR-1246, miR-150-5p, miR-21-5p, miR-223-3p and miR-23a-3p) in EVs as early diagnostic biomarkers for CRC. Compared with healthy controls, these miRNAs were significantly elevated in CRC patients [98]. In addition, they were significantly upregulated even in early-stage CRC patients and decreased after surgical resection. Recently, Wang et al. reported the potential of miR-125a-3p in plasma-derived EVs as a biomarker for early-stage colon cancer. They selected candidate miRNAs by small RNA sequencing and validated them in 50 early-stage CRC patients and 50 matched healthy volunteers. Although the diagnostic power of miR-125a-5p by itself was not very high (AUC: 0.6849), the multivariate model showed an increased diagnostic power in combination with CEA with an AUC of 0.855, indicating that miR-125a-3p is an independent biomarker from CEA [99].

Despite surgical intervention and adjuvant therapy, recurrence is common in patients with CRC [100]. Several studies have shown that miRNAs in EVs could be potential biomarkers of recurrence in CRC. Matsumura et al. revealed that miR-19a-3p in EVs could be a prognostic biomarker for recurrence in CRC patients. They performed miRNA microarray analysis of EVs from patients with recurrence and with non-recurrence. They also performed microarray and CGH array analysis in 124 CRC tissue samples. By comparing these results, they selected the miR-17-92a cluster as candidate miRNAs and validated them by qRT-PCR. Finally, they showed an association between the expression of miR-19a-3p in serum-derived EVs and a poorer prognosis [101]. In 2016, Liu et al. showed that the level of miR-4772-3p in EVs is significantly reduced in patients with stage II/III CRC patients with recurrence, with an AUC of 0.72, sensitivity of 78.6% and specificity of 77.1%. In that study, RNA seq-based miRNA profiling methods were performed to select candidate miRNAs [102].

### Prostate cancer

Prostate cancer (PCa) is the most frequently diagnosed male tumor and the third leading cause of cancer-related death in males in the United States [89]. Prostate-specific antigen (PSA) is the gold standard biomarker to diagnose and monitor the response to treatment. However, PSA has a low specificity with a high false positivity in patients with benign prostatic hyperplasia (BPH) [103]. Therefore, new biomarkers are needed for the accurate diagnosis and stratification of patients with PCa.

Bryant et al. analyzed miRNAs in plasma-derived EVs from a cohort of 78 PCa patients and 28 normal control individuals using a microarray panel of 742 miRNAs, finding a total of 12 differentially quantified miRNAs. Among these miRNAs, they confirmed an association of miR-141-3p and miR-375 with metastatic PCa using serum-derived EVs in a separate cohort by qRT-PCR [104]. miR-141-3p is one of the most common cancer-associated miRNAs, which is useful for the diagnosis of prostate cancer and prediction of metastasis [105, 106]. Li et al. reported the effectiveness of miR-141-3p in EVs by comparing PCa with BPH patients and healthy controls [107]. They also found that the expression level was higher in metastatic PCa patients compared with localized patients, with an AUC of 0.8694, sensitivity of 80% and specificity of 87.1% [107]. Due to the anatomical localization of the prostate, urine appears to be an ideal substrate to detect prostate carcinogenesis. Digital rectal examination (DRE) enhances the analytical performance of biomarker analysis in EVs, so a popular time for urine collection is after DRE [108]. Foj et al. recently revealed that among the most commonly deregulated miRNAs (miR-21-5p, miR-141-3p, miR-375, miR-214-3p and let-7c-5p) in PCa patients [105, 109, 110], miR-21-5p, miR-375 and let-7c-5p are significantly upregulated in urinary EVs from PCa patients compared with healthy controls [111].

Huang et al. described prognostic biomarkers for patients with advanced-stage prostate cancer. They performed RNA sequencing to identify candidate miRNAs in the screening cohort (n = 23) and confirmed that miR-1290 and miR-375 in EVs are significantly associated with poor overall survival in castration-resistant prostate cancer (CRPC) patients (n = 100) [112].

The recent clinical introduction of novel antiandrogens and chemotherapeutics has extended the survival of patients with metastatic CRPC (mCRPC). However, the rates of de novo and acquired resistance are high, and thus a liquid biopsy that can rapidly, sensitively and robustly identify which patients will respond to the treatment is required [113]. The androgen receptor splice variant 7 (AR-V7) is associated with resistance to hormonal therapy in mCRPC, and many researchers are focusing on the possibility of AR-V7 as a biomarker [114, 115]. Although this report is not focused on miRNA, Del et al. demonstrated AR-V7 detected in RNA extracted from EVs could be a predictive biomarker of resistance to hormonal therapy [116].

### Breast cancer

Breast cancer is the most common cancer in women in the United States, with an estimated 252,710 cases diagnosed in 2017 [89]. Traditional diagnostic methods, such as mammography, are effective but are known to have limited specificity and sensitivity. Although many studies have focused on the detection of circulating miRNAs in the serum or plasma of breast cancer patients, only a few studies to date have reported miRNAs in EVs from breast cancer patients. Eicheler et al. revealed that miR-101-3p and miR-372-3p in serum-derived EVs are significantly different between patients with breast cancer and healthy volunteers. They selected candidate miRNAs (miR-101-3p, miR-372-3p and miR-373-3p) based on previous reports and quantified the expression of these miRNAs in serum-derived EVs from breast cancer patients (n = 50) and healthy volunteers (n = 12) by qRT-PCR [117]. In another study, Hannafon et al. performed small RNA-seq and selected several miRNAs (miR-1246 miR-21-5p, miR-122-5p and let-7a-5p) that were enriched in EVs derived from a breast cancer cell line compared with an epithelial cell line. They subsequently validated the expression of miRNAs in plasma-derived EVs and showed significantly higher levels of miR-1246 and miR-21-5p in breast cancer patients (n = 16) compared with healthy controls (n = 16), each with AUCs of 0.69 and a combined AUC of 0.73 [118].

### Ovarian cancer

Ovarian cancer ranked fifth in cancer deaths among women in 2017 and is the leading cause of death among gynecological malignancies in the United States [89]. EOC account for 90% of ovarian cancer, and its high mortality may be related to the asymptomatic status of affected individuals until the late stage of disease, and therefore is detected too late. Currently, CA125 is the most frequently used serum biomarker, but it is not sufficiently specific for the diagnosis of EOC at an early stage [119].

Meng et al. revealed that miR-373-3p, miR-200a-3p, miR-200b-3p and miR-200c-3p in EVs are useful to distinguish malignant and benign ovarian disease. They selected six candidate miRNAs (miR-141-3p, miR-373-3p, miR-200a-3p, miR-200b-3p, miR-200c-3p and miR-429 from previous reports and evaluated them by qRT-PCR. They also evaluated the prognostic miRNAs in EVs and found that miR-200b-3p and miR-200c-3p were associated with an advanced FIGO stage and lymph node metastasis [120]. Ovarian cancer cells aggressively spread to the peritoneal cavity; therefore, ascetic fluid could be a useful and critical biomarker for ovarian cancer. Recently, Yokoi et al. found that highly metastatic ovarian cancer cells secrete EVs carrying MMP1 mRNA, and they revealed the potential utility of ascetic EVs as a risk indicator of peritoneal metastasis [69]. Although we could not find a clinical study to demonstrate the usefulness of miRNAs in ascites-derived EVs in ovarian cancer, as Tokuhisa et al. reported their effectiveness in gastric cancer [121], miRNAs in ascetic fluid hold great possibilities for predicting the status of ovarian cancer patients.

### Melanoma

Melanoma is the most deadly form of skin cancer [89]. Localized melanoma is curable by surgical resection, and the 5-year survival rate is 97%. However, once the melanoma has spread to distant organs, it is refractory to existing therapies, and the 5-year survival rate declines to approximately 10% [122]. S100B, melanoma inhibitory activity (MIA) and lactate dehydrogenase (LDH) are the most widely used biomarkers for the metastatic developmental stage of melanoma, but they have very low sensitivity [123]. Thus, it is important to identify novel biomarkers for the management of melanoma patients.

Although several diagnostic EV biomarkers of melanoma have been reported [76, 123, 124], only two reports have been published concerning the effectiveness of miRNA in EVs as biomarkers. In 2014, Alegre et al. first assessed the diagnostic role of miRNAs in EVs derived from melanoma [125]. They selected miR-125b-5p, the circulating level of which had been assessed in previous reports of several kinds of cancers other than melanoma [126, 127]. In their study, miR-125b-5p in serum EVs was significantly suppressed in advanced melanoma patients compared with healthy controls [125]. In 2015, Pfeffer et al. performed a microarray analysis using RNA prepared from plasma-derived EVs and validated the candidate miRNAs. They found that miR-17-5p, miR-19a-3p, miR-21-5p, miR-126-3p and miR-149-5p are expressed at higher levels in plasma-derived EVs from metastatic melanoma patients in comparison to normal control volunteers [128].

### Limitations for diagnostic applications

As we have shown, a number of studies have focused on the clinical utility of miRNAs in EVs as tumor biomarkers (Table 2). Although some miRNAs in EVs are also highly expressed in serum or tissue, not all have provided consistent results, even in the same cancer types. One of the main explanation may be due to differences in cohort composition. To date, almost all studies have examined only a limited number of samples. miRNA levels correlate not only with disease condition but also, to some extent the characteristics of the patients, such as age, sex and ethnicity. Therefore, if the number of samples is limited, such a characteristic difference may cause inconsistent results between studies, and the lack of reference data to set up treatment thresholds. To overcome this problem, initial large-scale inter-laboratory studies should be performed.

**Table 2:** Potential use of miRNAs inEVs as cancer biomarkers

Cancer type	Role	miRNAs in EVs	Source	Isolation method	Type assay	Normalizing control	Findings	Refs.
Lung	Diagnostic	Screening model with miR-378a-3p, miR-379-5p, miR-139-5p and miR-200-5p	Plasma	ExoQuick	qRT-PCR	let-7a-5p	Distiguishing between control and patients with any kind of nodules (NSCLC and lung granuloma), with AUC of 0.908	[92]
	Diagnostic	Diagnostic model with miR-151-5p, miR-30a-3p, miR-200b-5p, miR-629-5p, miR-100-5p and miR-154-3p	Plasma	ExoQuick	qRT-PCR	let-7a-5p	Distiguishing between NSCLC and lung granuloma, with AUC of 0.76	[92]
	Diagnostic	Panel with let-7-5p, miR-21-5p, miR-24-3p and miR-486-5p	Plasma	Ultracentrifugation + immunoaffinity magnetic beads (anti EpCAM)	RNA seq, qRT-PCR	Cel-miR-39-3p	Distiguishing NSCLC from symptomatic set, with AUC of 0.899	[93]
	Diagnostic	Panel with miR-181-5p, miR-30a-3p, miR-30e-3p and miR-361-5p	Plasma	Ultracentrifugation + immunoaffinity magnetic beads (anti EpCAM)	RNA seq, qRT-PCR	Cel-miR-39-3p	Distinguishing adenocarcinoma from symptomatic set, with AUC of 0.936	[93]
	Diagnostic	Panel with miR-10b-5p, miR-15b-5p, and miR-320b	Plasma	Ultracentrifugation + immunoaffinity magnetic beads (anti EpCAM)	RNA seq, qRT-PCR	Cel-miR-39-3p	Distinguishing SCC from symptomatic set, with AUC of 0.911	[93]
	Prognostic	miR-23b-3p, miR-10b-5p and miR-21-5p	Plasma	ExoQuick	qRT-PCR, qRT-PCR	let-7a-5p	Associating with poor OS	[94]
	Prognostic	miR-21-5p and miR-4257	Plasma	Ultracentrifugation	Microarray, qRT-PCR	miR-16-5p	Associating with poor DFS	[95]
Colorectal	Diagnostic	let-7a-5p, miR-1229-3p, miR-1246, miR-150-5p, miR-21-5p, miR-223-3p and miR-23a-3p	Serum	Ultracentrifugation	Microarray, RT-PCR	miR-451a	Comparing primary CRC with healthy control	[98]
	Diagnostic	miR-125a-3p	Plasma	ExoQuick	RNA seq, qRT-PCR	miR-30e-5p	Distinguishing CRC from control, with AUC of 0.6849. Combination of miR-125a-3p with CEA improve the AUC 0.855	[99]
	Prognostic	miR-19a-3p	Serum	Ultracentrifugation or Total Exosome Isolation Kit	Microarray, qRT-PCR	miR-16-5p	Associating with poor OS and poor DFS	[101]
	Prognostic	miR-4772-3p	Serum	ExoQuick	RNA seq, qRT-PCR	miR-16-5p	Distinguishing recurrent CRC from non recurrent, with AUC of 0.72	[102]
Prostate	Diagnostic	miR-141-3p and miR-375	Serum	ExoMir extraction kit	Microarray, qRT-PCR	Cel-miR-39-3p	Comparing recurrent (metastatic) patients with non-recurrent patients	[104]
	Diagnostic	miR-141-3p	Serum	ExoQuick	qRT-PCR	Cel-miR-39-3p	Comparing PCa with BPH and control	[107]
	Diagnostic	miR-21-5p, miR-375 and let-7c-5p	Urine	Ultracentrifugation	qRT-PCR	Cel-miR-39-3p	Comparing Pca patients with healty controls AUCs: miR-21, 0.713; miR-375, 0.799; let-7c, 0.679	[111]
	Prognostic	Combination of miR-1290 and miR-375	Plasma	ExoQuick	RNA seq, qRT-PCR	Genomic mean of miR-30a-5p and miR-30e-5p	Associating with poor OS	[112]
Breast	Diagnostic	miR-101-3p and miR-372-3p	Serum	ExoQuick	qRT-PCR	Mean value of miR-16-5p and miR-484	Comparing invasive breast cancer patients with healthy control	[117]
	Diagnostic	Combination of miR-1246 and miR-21-5p	Plasma	Ultracentrifugation or ExoQuick	RNA seq, qRT-PCR	Cel-miR-54-3p	Distinguishing breast cancer patients with healthy control, with AUC of 0.7266	[118]
Ovarian	Diagnostic	miR-373-3p, miR-200a-3p, miR-200b-3p and miR-200c-3p	Serum	Total exosome isolation reagent	qRT-PCR	miR-484	Comparing EOC with healthy control	[120]
	Prognostic	miR-200a-3p, miR-200b-3p and miR-200c-3p	Serum	Total exosome isolation reagent	qRT-PCR	miR-484	Associating with advanced FIGO stage and lymph node metastasis	[120]
Melanoma	Diagnostic	miR-125-5p	Serum	ExoQuick	qRT-PCR	miR-16-5p	Comparing metastaitic melanoma patients with disease free patients with melanoma and helthy conrtols	[125]
	Diagnostic	miR-17-5p, miR-19a-3p, miR-21-5p, miR-126-3p, miR-149-5p	Plasma	ExoQuick	Microarray, qRT-PCR	Cel-miR-39-3p	Comparing metastatic melanoma patients with healthy controls	[128]

In addition, several technical obstacles and scientific topics should also be considered. For effective biomarker analysis of EVs, a standardized method of EV collection is needed. As mentioned above, we currently cannot completely distinguish each type of EV. Ultracentrifugation is most common and conventional way to collect EVs, but it takes a large amount of time. Exo Quick is a fast and simple procedure, but it is a relatively crude isolation method with contaminating soluble proteins [9]. Density gradient-based isolation using sucrose or iodixanol (OptiPrep™) can be used to isolate each EV fraction with greater purity than other methods. However, its application of the density gradient method in the clinical setting is questionable due to complications [129]. Furthermore, the differences in pre-analytical variables, such as sample-collecting and storage protocols are also important [130]. Therefore, the establishment of a standardized method that is simple, rapid, and has a high sensitivity of the sample purify is required.

Current miRNA measurement techniques should also be considered. Many studies have selected candidate miRNAs in EVs based on microarray results and validated them by qRT-PCR. qRT-PCR requires a suitable reference control gene that is challenging to identify. Some reports have used miR-16-5p as a housekeeping gene, which may serve as one standard [101, 105]. However, recent investigations of serum miRNA revealed a high variability in some diseases [131, 132]. In addition, the spike-in external control, such as *C. elegans* miR-39-3p, is also a popular normalization control, but it is difficult to completely regulate the amount of this artificial external control added to different samples. The use of non-suitable reference genes may impede our understanding of the expression levels of miRNAs, and thus further cooperative studies are needed to identify proper housekeeping miRNAs.

A procedure to select candidate miRNAs is another scientific topic. In some reports, candidate miRNAs were selected by referencing previous findings for serum or plasma circulating miRNAs; however, miRNA profiles in EVs and circulating miRNA profiles were not completely consistent [133]. Microarray provides a genome-wide expression profile of miRNAs, facilitating the detection of a large number of aberrant miRNAs. However, probe development is challenging for some miRNAs [134]. Some recent reports selected candidate miRNAs by RNA-seq [93, 99, 102, 112, 118]. The advantage of using RNA-seq technology is its provision of a comprehensive analysis of the whole transcriptomes and distinction between miRNAs that differ by even 1 nucleotide. Compared with microarray technology, RNA-seq has the advantage of higher sensitivity and the ability to detect new miRNAs that have not been previously reported [135]. In addition, miRNAs show sequence heterogeneity at the 3′ and 5′ ends. This variation, which is called isomiR, is difficult to detect particularly using qPCR-based methods and is also exhibited in EVs [136]. Furthermore, several recent studies have focused on non-coding RNAs other than miRNAs in EVs [59, 137]. Therefore, to detect these RNAs, the demand for high-throughput technologies such as RNA-seq will increase.

Recently, it was revealed that circulating miR-17-5p had been found at elevated level in all cancer types studied, and referred as early alarm signal for cancer [138]. Although miR-17-5p is not specific for a single type of cancer, combinations of miR-17-5p and cancer specific miRNAs in EVs might provide additional diagnostic power.

Thus, although miRNAs in EVs have great potential as cancer diagnostic or prognostic biomarkers, more investigations are required for effective implementation in the clinical setting.

## Conclusion

In this review, we summarize current EV research to discuss the possibility of using the miRNAs in EVs in clinical applications. EVs play a pivotal role in the regulation of multiple systemic pathophysiological processes. Thus, targeting intercellular communication will provide a new therapeutic strategy. In addition, compared with biomarkers detected in conventional specimens such as serum, plasma or urine, EV biomarker provide comparable or higher specificity and sensitivity due to their stability. Furthermore, there has been intense interest in the potential of EVs as delivery vehicles, because EVs have some great characteristics, such as stability in the blood circulation, low side effect, and tropism to some organs [139]. Until now, several articles revealed that administration of exogenous EVs including miRNA or siRNA can be therapeutic strategies [140, 141].

Although further research and development are needed for implementation in the clinical setting, the clinical utility of EVs is promising. EV research is developing quite rapidly, and thus we may be able to use them as therapeutic tools in the near future.

We ardently hope that the advancements in EV research will contribute to the treatment of cancer.

## Abbreviations

EV: extracellular vesicle
miRNA: microRNA
mRNA: messenger RNA
MVB: multivesicular body
ESCRT: endosomal-sorting complex required for transport
Hsp: heat shock protein
ISEV: international society of extracellular vesicles
3′ UTR: 3′ untranslated region
SOCS5: suppressor of cytokine signaling 5
nSMase2: neutral sphingomyelinase 2
TIMP-1: tissue inhibitor of metalliprotease-1
ZO-1: zonula occludens-1
BBB: blood brain barrier
PDPK1: 3-phosphoinositide-dependent protein kinase-1
CAF: cancer-associated fibroblast
EMT: epithelial–mesenchymal transition
TNBC: triple negative breast cancer
ECM: extracellular matrix
MDSC: myeloid-derived-suppressor cell
TLR: toll-like receptor
TAM: tumor-associated macrophage
EOC: epithelial ovarian cancer
PD-L1: programmed death-1 ligand
CLL: chronic lymphocytic leukemia
MET: mesenchymal-to-epithelial transition
NSCLC: non-small-cell lung cancer
SOCS3: suppressor of cytokine signaling 3
BM–MSC: bone marrow mesenchymal stem cell
MARCKS: myristoylated alanine-rich C-kinase substrate
SCC: squamous cell carcinoma
AUC: area under the receiving operating characteristic curve
CRC: colorectal cancer
PCa: prostate cancer
PSA: prostate-specific antigen
BPH: benign prostatic hyperplasia
CRPC: castration-resistant prostate cancer
mCRPC: metastatic CRPC
AR-V7: androgen receptor splice variant 7
LDH: lactate dehydrogenase
RNA-seq: RNA sequence
HSPG: heparan sulfate proteoglycan
GBM: glioblastoma
